# A Frequency Up-Conversion Piezoelectric Energy Harvester Shunted to a Synchronous Electric Charge Extraction Circuit

**DOI:** 10.3390/mi15070842

**Published:** 2024-06-28

**Authors:** Xuzhang Peng, Hao Tang, Zhongjie Li, Junrui Liang, Liuding Yu, Guobiao Hu

**Affiliations:** 1 Internet of Things Thrust, The Hong Kong University of Science and Technology (Guangzhou), Guangzhou 511400, China; xpeng842@connect.hkust-gz.edu.cn (X.P.); htang969@connect.hkust-gz.edu.cn (H.T.); guobiaohu@hkust-gz.edu.cn (G.H.); 2 School of Mechatronic Engineering and Automation, Shanghai University, Shanghai 200444, China; lizhongjie@shu.edu.cn; 3 School of Information Science and Technology, ShanghaiTech University, Shanghai 201210, China; liangjr@shanghaitech.edu.cn; 4 College of Power Engineering, Naval University of Engineering, No. 717 Jiefang Avenue, Wuhan 430033, China

**Keywords:** frequency up-conversion, equivalent circuit model, piezoelectric energy harvester, synchronous electric charge extraction

## Abstract

A frequency up-conversion piezoelectric energy harvester (FUC-PEH) consists of a force amplifier, a piezoelectric stack, a low-frequency oscillator (LFO), and a stop limiter. The force amplifier generates the amplification of stress on the piezoelectric stack. The LFO, comprising a spring and a mass block, impacts the stop limiter during vibration to induce high-frequency oscillations within the piezoelectric stack. In this paper, we represent and simplify the FUC-PEH as a lumped-parameter model based on piezoelectric material constitutive equations and structural dynamic theories. Using the electromechanical analogy, we developed an equivalent circuit model (ECM) of the FUC-PEH. A parametric study was performed to investigate the impact of system parameters, such as spring stiffness and concentrated mass, on the FUC-PEH performance. The collision-induced amplitude truncation (AT) effect enlarges the operation bandwidth. ECM simulations show that low-frequency input excitation is converted into a high-frequency output response, enhancing the energy conversion efficiency. Furthermore, we aimed to improve the FUC-PEH’s performance using a synchronous electric charge extraction (SECE) circuit. Using the ECM approach, we established a system-level model that considers the electromechanical coupling behavior. The simulation results provide insights into the performance of FUC harvesters with SECE circuits and offer valuable design guidance.

## 1. Introduction

An energy harvester can harness kinetic energy from the ambient environment [1,2,3]. Piezoelectric energy harvesters (PEHs) are one of the main technologies for collecting vibration energy, offering a sustainable power solution for low-power electronics [4,5]. However, the bandwidth of traditional piezoelectric energy harvesters is limited, and the performance of PEHs decreases dramatically if the ambient vibration frequency deviates from the resonant frequency. Therefore, many efforts have been devoted to widening the bandwidth of PEHs. The proposed multi-stable [6,7,8], multi-degree-of-freedom structures [9,10] and frequency up-conversion (FUC) mechanism [11] are standard approaches for broadband energy harvesting.

The multi-stable PEHs improve the output performance by utilizing the large amplitude inter-well vibrations [12,13]. Norenberg et al. [14] and Liu et al. [15] investigated the impact of bistable structures on the energy-harvesting performance of piezoelectric cantilever beams. Researchers further explored the tri-stable [16,17] and quad-stable [18,19] configurations to reduce the hindrance of potential barriers for weak excitation. However, the implementation of steady states escalates the structural complexity and decreases the system’s robustness. The performance improvement of multi-degree-of-freedom PEHs can be achieved through a simplified structure [20,21,22]; nevertheless, these studies have not considered the impact of spatial constraints on the output performance. Meanwhile, most ambient vibration is distributed within an ultra-low-frequency range. The PEHs with high resonant frequencies pose a challenge when aligning with low-frequency vibration excitation in practical application scenarios.

The exploration of FUC mechanisms has shown the ability to enhance the output of PEHs by extending the bandwidth and decreasing the resonant frequency [23,24,25]. For instance, Zhang et al. [26] proposed an impact-and-rope-driven PEH, resulting in a bandwidth from 2.5 Hz to 10 Hz by adjusting the rope margin. Panthongsya et al. [27] developed an FUC-PEH based on springs and permanent magnets and significantly improved the energy conversion efficiency by establishing an array of the PEHs. Pietro et al. [28] studied the effects of tip mass and impact position on the output power of an impact-based FUC-PEH. Wu et al. [29] realized a relatively high output power of an FUC-PEH under ultra-low-frequency vibration through the internal resonance mechanism. Moreover, hybrid designs have emerged, in which energy harvesters incorporating FUC structures have demonstrated a high-power output by leveraging both piezoelectric and electromagnetic mechanisms [23,30].

Nevertheless, effectively harnessing the FUC mechanism is not devoid of challenges. A common hurdle associated with many FUC mechanisms is dynamic nonlinearities introduced by collision phenomena, which pose significant challenges in modeling and analyzing the FUC-PEHs. It is imperative to develop models that solve the complex dynamics induced by collisions to optimize the energy harvesting performance of FUC-PEHs.

Additionally, an interface circuit is needed to realize the AC–DC conversion and extract power from the FUC-PEHs. Traditionally, the standard circuit (SEH) [31] is used. Subsequently, some nonlinear circuits have been proposed, like P-SSHI [32], S-SSHI [33], and SECE [34] topologies, to enhance the energy harvesting efficiency further. However, the applications of those circuits may present greater challenges in control during the synchronous instant in which they are shunted to the FUC-PEHs with damped waveforms.

Considering these challenges, this paper delineates an equivalent circuit model (ECM) to capture the dynamics and predict the performance of an FUC-PEH. The proposed harvester comprises a force amplifier, a piezoelectric stack, a low-frequency oscillator (LFO), and a stop limiter. This paper first demonstrates how to represent and simplify the FUC-PEH as a lumped-parameter model based on structural dynamic theories. Then, drawing on the electromechanical analogy, we develop an ECM of the FUC-PEH. A subsequent parametric study investigates the influences of system parameters, such as spring stiffness and proof mass, on the performance of the FUC-PEH. Finally, a self-powered synchronous electric charge extraction (SP-SECE) circuit is implemented to harvest energy from the proposed FUC-PEH further to enhance the performance of the entire electromechanical system. Compared with the SEH circuit, the SP-SECE circuit exhibited better capacity in terms of both charging speed and charged power.

## 2. Materials and Methods

**Figure 1:**
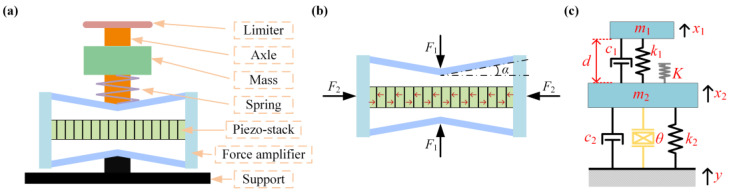
(**a**) The structural configuration of the FUC-PEH; (**b**) the operation principle of the force amplifier; (**c**) the schematic of the equivalent lumped 2DoF model.

In this study, we present a structural configuration of an FUC-PEH, depicted in Figure 1a. The proposed harvester primarily comprises a force amplifier, a piezoelectric stack, a low-frequency oscillator (LFO), and a stop limiter. The piezoelectric stack, composed of multiple piezoelectric units in parallel, possesses a rigid and stiff structure, rendering it suitable for operation within high-frequency ranges instead of the low frequencies of ambient vibrations. The force amplifier can convert the external excitation force to larger stress on a piezoelectric stack secured in the center of the amplifier [35,36]. The operation principle of a piezoelectric stack with a force amplifier is illustrated in Figure 1b. Red arrows indicate the polarization direction of each layer of the stack. The force amplification relationship can be derived as $F_{2}=\cot\alpha \cdot F_{1}$, where *α* is the intersection angle of the connecting beam concerning the horizontal direction, and *F*_1_ and *F*_2_ are the external force on the amplifier and the applied force on the stack, respectively [37,38].

The LFO, encompassing an elastic spring and a mass block, impacts the stop limiter during the vibration process. The limiter applies the impact force to the amplifier through the axle, thereby inducing oscillations within the piezoelectric stack. During oscillations, the force amplifier generates micro-elastic deformations, amplifying the stress and strain experienced by the piezoelectric stack. The force amplifier, axle, stop limiter, and piezoelectric stack form an equivalent spring-mass structure, thereby introducing an additional degree of freedom (DoF).

The harvester can be abstracted and represented as a corresponding equivalent lumped-parameter model, illustrated in Figure 1c. Herein, *m*_1_ symbolizes the mass block, while *m*_2_ represents the equivalent mass encompassing the axle, stop limiter, force amplifier, and piezoelectric stack. The parameters *k_i_* and *c_i_* (*i* = 1, 2) denote the equivalent stiffness and damping coefficients, respectively. Note that the subscripts 1 and 2 distinctly signify the degrees of freedom associated with *m*_1_ and *m*_2_. The governing equations of the FUC-PEH can be articulated as follows:

(1) $$\left\{\begin{array}{l} m_{1}{\ddot{u}}_{1}\left(t\right)+c_{1}\left({\dot{u}}_{1}\left(t\right)-{\dot{u}}_{2}\left(t\right)\right)+k_{1}\left(u_{1}\left(t\right)-u_{2}\left(t\right)\right)+I\left(u_{1}\left(t\right),u_{2}\left(t\right)\right)=-m_{1}\ddot{y}\left(t\right)\\ m_{2}{\ddot{u}}_{2}\left(t\right)+c_{2}{\dot{u}}_{2}\left(t\right)+k_{2}u_{2}\left(t\right)+c_{1}\left({\dot{u}}_{2}\left(t\right)-{\dot{u}}_{1}\left(t\right)\right)+k_{1}\left(u_{2}\left(t\right)-u_{1}\left(t\right)\right)-I\left(u_{1}\left(t\right),u_{2}\left(t\right)\right)+\theta v\left(t\right)=-m_{2}\ddot{y}\left(t\right)\\ \frac{v\left(t\right)}{R_{l}}+C_{p}\dot{v}\left(t\right)=\theta {\dot{u}}_{2}\left(t\right) \end{array}\right.,$$

in which *u_i_* (*i* = 1, 2) denotes the displacements of the equivalent masses *m*_1_ and *m*_2_ in relation to the base. Further, *y* represents the base excitation displacement. At the same time, *v*, *R*, *C_p_*, and *θ* correspond to the output voltage, the external load resistance, the capacitance of the piezoelectric stack, and the equivalent electromechanical coupling coefficient, respectively.

This study employed a simplification approach to compute the equivalent spring stiffness *k*_2_, as calculated by $k_{2}=2E_{s}A_{s}\sin^{2}\alpha /l$ [39]. The parameters *E_s_*, *A_s_*, and *l* distinctly characterize the Young’s modulus, cross-sectional area, and length of the connecting rod within the force amplifier. The multi-layer piezoelectric stack is represented as an equivalent single piezoelectric block in this study. The equivalent electromechanical coupling coefficient *θ* is determined by $\theta =ne_{33}A/L$ [34,40]. Herein, the parameters *n*, *e*_33_, and *A,* respectively, signify the number, piezoelectric coefficient, and section of the unit piezoelectric element. *L* denotes the length of the piezoelectric stack. Subsequently, the finite element simulation software COMSOL Multiphysics 6.1 was employed to validate the equivalent material properties of the equivalent single piezoelectric block. In the finite element simulation, we constructed a piezoelectric stack model consisting of ten piezoelectric units and a piezoelectric block model with identical geometric dimensions. The determination of the equivalent material properties for the piezoelectric block is referenced in [40]. Both the finite element models were fixed at one end, and a uniform load of 10 N/m^2^ was applied at the other end while maintaining a consistent mesh division mode. The voltages of the piezo-stack and equivalent piezo-block in static analysis were measured as 0.19401 mV and 0.19737 mV, respectively. The observed discrepancy between the equivalent and actual parameters was merely 1.74%.

The initial distance between *m*_1_ and *m*_2_, denoted as *d*, was observed when the FUC-PEH was in a quasi-static state under the influence of gravity. The impact between the LFO and the stop limiter occurred due to *m*_1_ and *m*_2_ being positioned within a relative distance less than *d*. A piecewise linear function characterizes the impact-induced nonlinear force as follows:

(2) $$I\left(u_{1}\left(t\right),u_{2}\left(t\right)\right)=\left\{\begin{array}{cc} K\left(u_{1}\left(t\right)-u_{2}\left(t\right)+d\right) & u_{1}\left(t\right)-u_{2}\left(t\right)<-d\\ 0 & u_{1}\left(t\right)-u_{2}\left(t\right)\geq -d \end{array}\right.,$$

where *K* stands as a representation of the collision stiffness during the impact phase.

By defining the following parameters: $x_{1}=u_{1},\;x_{2}={\dot{u}}_{1},\;x_{3}=u_{2},\;x_{4}={\dot{u}}_{2},\;x_{5}=v$, Equations (1) and (2) can be rearranged in the state space form as follows:

(3) $$\left\{\begin{array}{l} x_{2}\\ -\frac{1}{m_{2}}\left[m_{1}\ddot{y}+c_{1}\left(x_{2}-x_{4}\right)+k_{1}\left(x_{1}-x_{3}\right)+I\left(x_{1},x_{3}\right)\right]\\ x_{4}\\ -\frac{1}{m_{2}}\left[m_{2}y+c_{2}x_{4}+k_{2}x_{3}+c_{1}\left(x_{4}-x_{2}\right)+k_{1}\left(x_{3}-x_{1}\right)-I\left(x_{1},x_{3}\right)+\theta x_{5}\right]\\ \frac{1}{C_{p}}\left[\theta x_{4}-\frac{x_{5}}{R_{l}}\right] \end{array}\right.,$$

(4) $$I\left(x_{1},x_{3}\right)=\left\{\begin{array}{ll} K\left(x_{1}-x_{3}+d\right) & x_{1}-x_{3}<-d\\ 0 & x_{1}-x_{3}\geq -d \end{array}\right.,$$

By employing Equations (3) and (4), the numerical solution for the voltage response of the FUC-PEH can be derived by utilizing MATLAB R2023b.

The collision phenomena enhance the performance of the FUC-PEH in the low-frequency range by widening the operational bandwidths. However, the collision phenomena introduce nonlinearity in the dynamics, thus complicating the system analysis. To this end, we propose an equivalent circuit modeling method thoroughly considering the electromechanical coupling behavior for system-level simulation analyses. According to electromechanical analogies, the mechanical quantities are equivalent to the electrical quantities, as detailed in Table 1. Based on these analogies, the governing equations in Equation (1) can be reformulated as Equation (5).

(5) $$\left\{\begin{array}{l} L_{1}{\ddot{q}}_{1}+R_{1}\left({\dot{q}}_{1}-{\dot{q}}_{2}\right)+\frac{1}{C_{1}}\left(q_{1}-q_{2}\right)+I\left(q_{1},q_{2}\right)=-L_{1}\ddot{y}\left(t\right)\\ L_{2}{\ddot{q}}_{2}+R_{2}{\dot{q}}_{2}+\frac{1}{C_{2}}q_{2}+R_{1}\left({\dot{q}}_{2}-{\dot{q}}_{1}\right)+\frac{1}{C_{1}}\left(q_{2}-q_{1}\right)-I\left(q_{1},q_{2}\right)=-L_{2}\ddot{y}\left(t\right)\\ \frac{v\left(t\right)}{R}+C_{p}\dot{v}\left(t\right)=N{\dot{q}}_{2}\left(t\right) \end{array}\right.,$$

**Figure 2:**
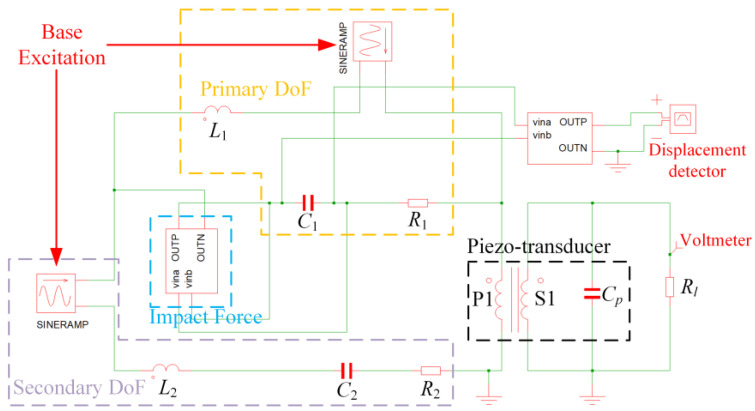
Equivalent circuit model of the FUC-PEH.

According to Equation (5), an equivalent circuit model (ECM) of the FUC-PEH, as illustrated in Figure 2, was developed by harnessing the circuit simulation software SIMetrix-SIMPLIS 8.3. Within the ECM, the piezoelectric component can be signified as integrating a capacitor and an ideal transformer connected in parallel. Harmonic excitation is emulated using swept-sine voltage sources. The impact-induced nonlinear force can be generated by a defined arbitrary source. The part to the left of the transformer in Figure 2 represents the mechanical domain, whereas the right represents the circuit domain. The left-hand-side circuit was composed of two branches since the mechanical domain of the energy harvester was a 2DoF structure. One can measure the voltages across the capacitors and calculate the accumulated charges to ascertain the displacement *u*_1_. Moreover, direct measurements of the terminal voltage across the resistance can be obtained by placing a voltage probe.

**Figure 3:**
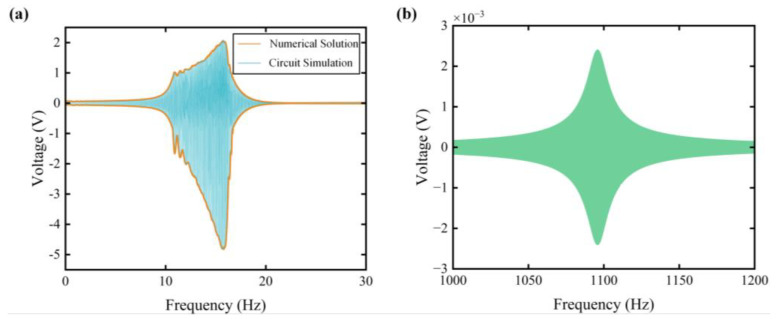
(**a**) Comparison of transient voltage responses from circuit simulation and numerical solution; (**b**) the voltage response of a PEH without the LFO in the frequency domain.

To validate the results derived from the equivalent circuit simulation, the numerical solution of Equation (1) was obtained for comparison. The system parameters of the FUC-PEH are listed in Table 2 and Table 3. Figure 3a compares the ECM simulation and numerical solution of voltage outputs in the frequency domain. For the simulation parameters, the excitation acceleration was gauged at 0.75 G, where G represents gravitational acceleration. Consequently, the result predicted from the circuit simulation almost overlapped the numerical solution. The operational bandwidth of the FUC-PEH showed substantial expansion, with an operational bandwidth (defined as the frequency span where the voltage reaches half of its peak amplitude) between 14.4 and 22.1 Hz both in the circuit simulation and the numerical solution. Note that the voltage curves were asymmetric; this is attributed to the single-sided impact between the LFO and the stop limiter. Hence, the ECM can be regarded as validated.

Furthermore, Figure 3b illustrates the voltage response of the FUC-PEH and a PEH without the LFO based on the equivalent circuit simulations. The impact-based frequency up-conversion mechanism significantly reduced the resonant frequency, decreasing from 1095.8 Hz to 15.75 Hz. Simultaneously, there was a substantial increase in the maximum voltage, rising from 2.4 mV to nearly 4.9 V. The results indicate that the FUC mechanism can effectively improve the output performance of the PEH. Regarding the operational bandwidth, we adopted a normalized parameter to fairly compare different configurations. The normalized operational bandwidth (NOB) is defined as follows:

(6) $$\mathrm{NOB}=\frac{\mathrm{operational}\;\mathrm{bandwidth}}{\mathrm{resonant}\;\mathrm{frequency}}.$$

The normalized operating behavior is a metric that considers the varying operating conditions of different structures. The NOB of the FUC-PEH was 0.4889, significantly higher than the NOB of the PEH without an LFO, which was 0.0196. Compared to the multi-stable PEH [22] with an NOB of 0.322 and the multi-degree-of-freedom PEH [15] with an NOB of 0.273, the proposed FUC mechanism exhibited a significant advantage.

## 3. Results and Discussion

### 3.1. Parametric Study

**Figure 4:**
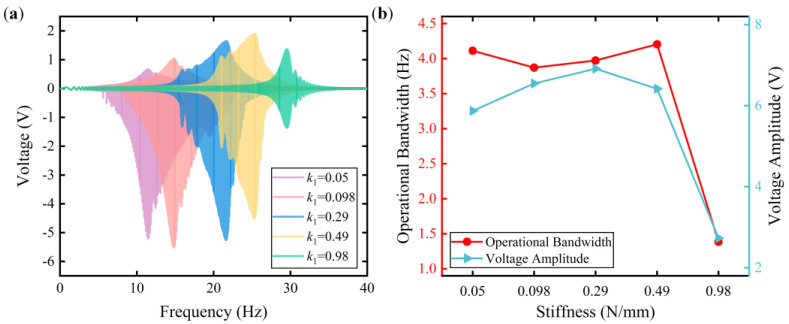
Voltage responses with different spring stiffnesses (0.05, 0.098, 0.29, 0.49, and 0.98 N/mm). (**a**) The frequency−domain voltage responses; (**b**) the operational bandwidth and voltage amplitude.

Employing the validated ECM, a parametric study was performed to investigate the influences of system parameters, such as the spring stiffness *k*_1_ and mass *m*_1_, on the performance of the FUC-PEH. Figure 4a depicts the frequency responses of the FUC-PEH with different spring stiffnesses, ranging from 0.05 to 0.98 N/mm with a mass of 30 g. For a spring stiffness of 0.98 N/mm, a symmetrical voltage profile was observed, and the peak voltage was significantly reduced relative to that for a lower spring stiffness. This phenomenon can be attributed to the increase in spring stiffness, which reduces the displacement amplitude of the mass block, thus preventing an impact collision of the mass block with the stop limiter. As the spring stiffness decreases, the resonances shift to lower-frequency domains, accompanied by asymmetrical voltage profiles. Under these conditions, the unilateral impact occurs in the harvester while the amplitude of the mass block is truncated. Attributable to the collision-induced amplitude truncation (AT) effect, the bandwidth of the FUC-PEH is enlarged.

To delve deeper into the effect of the spring stiffness on the performance, both the voltage amplitude (defined as peak-to-peak voltage) and the operational bandwidth were calculated for each case, which are displayed in Figure 4b. At a spring stiffness of 0.49 N/mm, the harvester had a maximum operational bandwidth of 4.2 Hz. The maximum voltage amplitude was 6.91 V when the spring stiffness was 0.29 N/mm. As the spring stiffness increased, the voltage amplitude increased, followed by a subsequent decline. Note that the tendency of the operational bandwidth diverged from that of the voltage amplitude. This difference might be due to the definition of the operational bandwidth. The operational bandwidth still decreased sharply beyond a spring stiffness of 0.49 N/mm.

**Figure 5:**
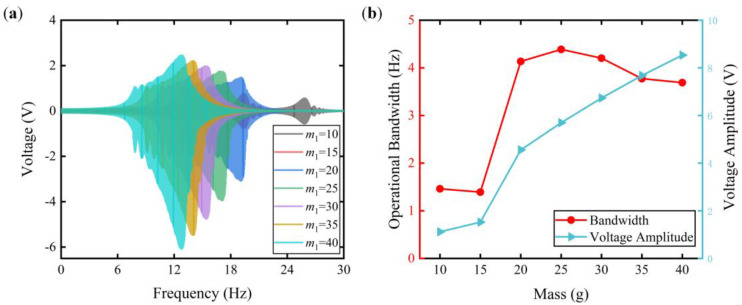
Voltage responses with different masses (10, 15, 20, 25, 30, 35, and 40 g). (**a**) The frequency−domain voltage responses; (**b**) the operational bandwidth and voltage amplitude.

Figure 5 delineates voltage responses across varying mass *m*_1_ values (10, 15, 20, 25, 30, 35, and 40 g), while maintaining the spring stiffness at 0.49 N/mm. As the mass increased, the resonances shifted to lower-frequency domains, as shown in Figure 5a. At masses of 10 and 15 g, the voltage profiles remained symmetric. This symmetry can be ascribed to the motion amplitude being insufficient for impact to occur, thereby preventing the AT effect. In Figure 5b, the voltage amplitude increased concomitant with an increment in mass. When the mass was 25 g, the maximum operational bandwidth was 4.39 Hz. Furthermore, the tendency of the operational bandwidth increase was followed by a subsequent decline. Therefore, a suitable proof mass and spring stiffness combination can yield an FUC-PEH with an enlarged operational bandwidth and enhanced voltage amplitude.

### 3.2. Integration of FUC-PEH and SECE Circuit

The SECE technique necessitates synchronous instants when the oscillator reaches its displacement or voltage extremums, which are realized by an external power source with a predefined frequency in most previous studies. However, the high oscillation frequency of the proposed FUC-PEH would complicate the control of this method. In this regard, a self-powered SECE (SP-SECE) topology with a self-detection function was adopted.

**Figure 6:**
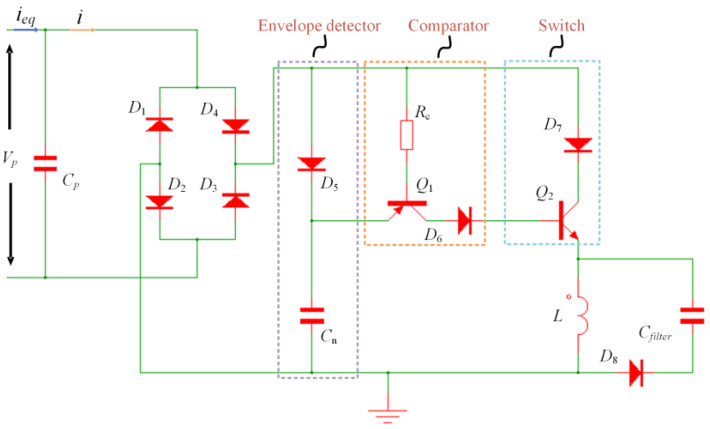
The topology of the SP-SECE circuit.

As shown in Figure 6, the SP-SECE circuit used an electronic breaker consisting of an envelope detector and a comparator. In the simulation, all diodes (D_1_ to D_8_) were the ideal model, and the transistors Q_1_ and Q_2_ were the 2N2904 and 2N2222 models, respectively, as provided in SIMetrix. A smaller inductor (470 μH) was selected to distinguish the synchronous instants from the high-frequency mechanical oscillations (around 1200 Hz) and the larger piezoelectric capacitor (1.732 μF). The values of C_n_ and R_b_ were selected as 50 nF and 6 kΩ to guarantee the validation of the electronic breaker at such a high frequency.

**Figure 7:**
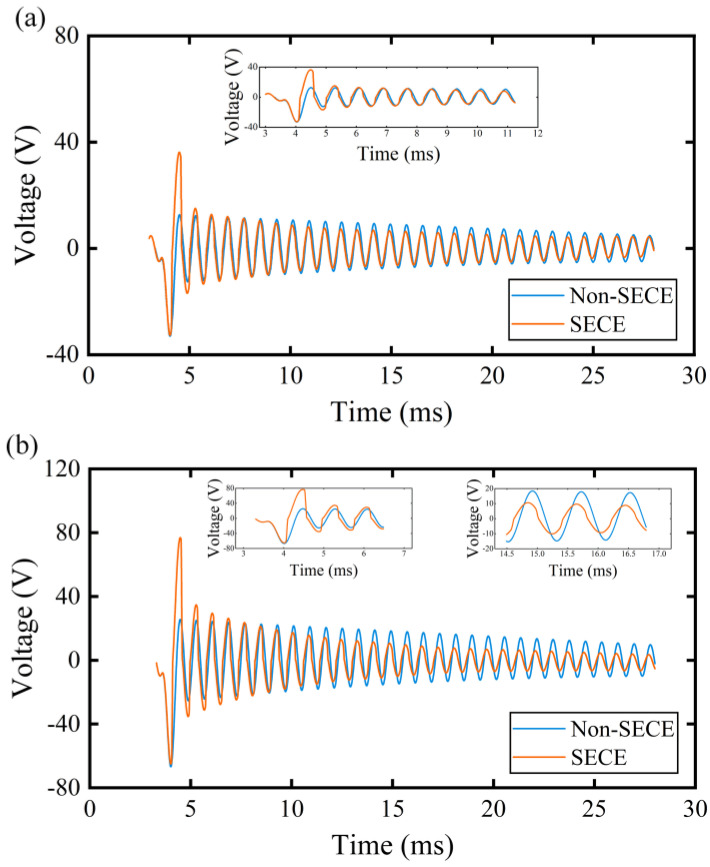
The waveforms of piezoelectric voltage with and without SP−SECE circuit under harmonic excitations. (**a**) acceleration: 2 G; (**b**) acceleration: 4 G.

Figure 7 displays the waveforms of the piezoelectric voltage under harmonic excitations at 2 G and 4 G accelerations alongside the original waveform in the open-circuit condition. It can be observed that the synchronous instant disappeared as the magnitude of piezoelectric voltage attenuated to around 10 V, indicating that the electronic breaker failed to conduct the LC oscillation in these cases. This phenomenon arises from the phase lag introduced by the SP-SECE topology. Indeed, the envelope capacitor C_n_ requires a specific charging duration to conduct Q_2_, during which the piezoelectric voltage would experience a specific drop corresponding to the decreasing displacement of the oscillator. In previous studies, the period of mechanical oscillation was significantly shorter than the charging duration, thus diminishing the influence of the phase lag. However, the voltage drop will be considerably enlarged when the oscillation frequency is extremely high. As a result, the voltage threshold for the synchronous instant is increased. In the enlarged view of Figure 7a, the disappearance of the synchronous instant occurred around the seventh peak of piezoelectric voltage. When the magnitude of acceleration increased to 4 G, the number of the synchronous instants also doubled to about fifteen, as specified in Figure 7b.

It is also observed that the magnitude of piezoelectric voltage was enlarged when the synchronous instant was distinct. The phenomenon may be attributed to the electromechanical coupling effect introduced by the SP-SECE circuit. It also suggests that the proposed piezoelectric stack refers to a weak electromechanical system since the SECE circuit performs much better in this case [41]. The enlargement effect will gradually degrade with briefer synchronous instants, and finally, the magnitude of the piezoelectric voltage would be smaller than the original counterpart in open-circuit conditions as the electronic breaker would bypass a small part of the current [42].

**Figure 8:**
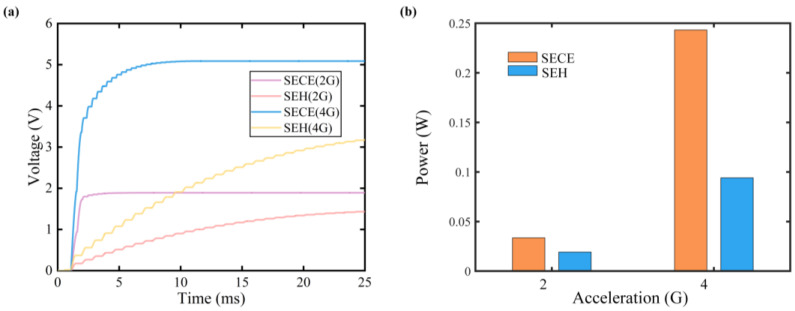
Charging performance of the SP-SECE and SEH circuits: (**a**) voltage; (**b**) power.

Figure 8 compares the charging performance of the SP-SECE and SEH circuits with a 470 μF capacitor over a period of approximately 24 ms, corresponding to a single-damped period of the FUC-PEH. The charging speed and charged voltage are shown in Figure 8a. It can be noticed that the charging process of the SP-SECE circuit ended at specific moments, which corresponded to the disappearance of the synchronous instant. In contrast, the SEH circuit allowed continuous charging during the whole period. Despite utilizing fewer waveforms for energy harvesting, the SP-SECE circuit achieved higher charged voltages and a significantly faster charging speed than the SEH circuit. Specifically, the charged voltages were 1.891 V and 1.431 V under 2 G acceleration and 5.087 V and 3.165 V under 4 G acceleration for the SP-SECE and SEH circuits. In terms of the charged power, the SP-SECE circuit harvested 1.75 times (33.6 mW and 19.2 mW) more energy than the SEH circuit under 2 G acceleration, as illustrated in Figure 8b. Benefiting from more triggered synchronous instants, the improvement increased to 2.58 times (243.2 mW and 94.2 mW) when the acceleration increased to 4 G. In addition, it can be inferred that the ratio of the charged power would further enlarge with longer charging times since a larger load voltage will raise the voltage threshold of the SEH circuit. Consequently, the SEH circuit can effectively utilize fewer waveforms for energy harvesting. On the other hand, the harvesting capacity of the SECE circuit would not be influenced by this factor, and therefore, the extracted energy is predicted to be the same in each damped period, suggesting that it may be more promising for energy harvesting using FUC harvesters.

Based on the above analysis, it can be concluded that the SP-SECE circuit has the potential to further enhance the performance of FUC-PEHs after the optimization of the mechanical structure and parameters. It is important to note that the SECE circuit only performs better in weak electromechanical coupling systems. Although the previous conclusions were drawn from the standard harmonic excitations, it is rational to assume that the coupling effect would still exist under damped excitation. Therefore, the performance of the SECE technique may still depend on the electromechanical coupling conditions for FUC-PEHs. Additionally, owing to the high frequency, nonlinearity, and large internal capacitance characteristics, the impedance matching of FUC-PEHs may be difficult to achieve when the SEH circuit is used. Therefore, the load independence of the SECE circuit is expected to remain beneficial in this case, and we will consider and discuss this issue in further research.

## 4. Conclusions

This paper develops an equivalent circuit model (ECM) for analyzing the performance of a frequency up-conversion piezoelectric energy harvester (FUC-PEH). The FUC-PEH, comprising a force amplifier, a piezoelectric stack, a low-frequency oscillator (LFO), and a stop limiter, enlarges the operational bandwidth due to impact-induced nonlinear force. Firstly, we simplify the FUC-PEH into a lumped-parameter model. Subsequently, drawing on the analogy between the mechanical and electrical domains, an equivalent circuit model is developed and substantiated through numerical solutions. Furthermore, we conducted a parametric study to investigate the influence of the spring stiffness and mass on the performance. It was discerned that a suitable combination of a proof mass and spring stiffness not only widens the operational bandwidth but also enlarges the voltage amplitude, a phenomenon caused by the collision-induced amplitude truncation effect. Finally, the further performance enhancement was validated by system-level simulations when the SP-SECE circuit was integrated with the proposed FUC-PEH; the feasibility and necessity for their integration design will be investigated in further study.

## Figures and Tables

**Table 1:** Analogies between the electrical domain and mechanical domain.

Equivalent Circuit Parameters	Mechanical Counterparts
Charge: *q*	Displacement: *u*
Current: $\dot{q}$	Velocity: $\dot{u}$
Inductance: *L_i_*	Effective mass: *m_i_*
Resistance: *R_i_*	Effective damping: *c_i_*
Capacitance: *C_i_*	Reciprocal of effective stiffness: 1/*k_i_*
Ideal transformer turn ratio: *N*	Electromechanical coupling: *θ*

**Table 2:** System parameters of the FUC-PEH.

Mechanical Parameters	Values
Effective mass *m*_1_ (g)	29.02
Effective mass *m*_2_ (g)	23.3
Effective stiffness *k*_1_ (N/m)	490
Effective stiffness *k*_2_ (N/m)	1.3756 × 10^6^
Damping coefficient *c*_1_ (Ns/m)	0.0422
Base excitation (m/s^2^)	7.35
Impact stiffness (N/m)	3000
Initial distance *d* (mm)	14.4
Load resistance *R_l_* (Ω)	1012
Damping coefficient *c*_2_ (Ns/m)	1.8916

**Table 3:** Parameters of the piezoelectric stack and the connecting rod.

	Description	Value
Piezoelectric stack	Number of layers *n*	180
	Piezoelectric constant *e*_33_ (10^−3^ Vm/N)	12.5
	Capacitance *C_p_* (μF)	1.732
	Cross-sectional area *A* (mm^2^)	25
	Length *L* (mm)	18
Rod in force amplifier	Intersection angle α (rad)	0.3
	Young’s modulus *E_s_* (GPa)	193
	Cross-sectional area *A_s_* (mm^2^)	12
	Length *l* (mm)	8.5

## Data Availability

The data supporting the findings of this paper are available from the corresponding authors on request.
